# Cannabinoid Hyperemesis Syndrome: A Case Report of Cyclic Severe Hyperemesis and Abdominal Pain with Long-Term Cannabis Use

**DOI:** 10.1155/2016/2815901

**Published:** 2016-11-17

**Authors:** Julia Hermes-Laufer, Lola Del Puppo, Ihsan Inan, François-Xavier Troillet, Omar Kherad

**Affiliations:** ^1^Department of Internal Medicine, Hôpital de La Tour, Avenue J.-D.-Maillard 3, 1217 Geneva, Switzerland; ^2^Department of Visceral Surgery, Clinique Générale Beaulieu, Chemin de Beau Soleil 20, 1206 Geneva, Switzerland; ^3^Department of Gastroenterology, Hôpital de La Tour, Avenue J.-D.-Maillard 3, 1217 Geneva, Switzerland; ^4^Faculty of Medicine, University of Geneva, Geneva, Switzerland

## Abstract

*Introduction.* Cannabinoid Hyperemesis Syndrome (CHS) is a rare condition that includes cyclic severe vomiting in subjects who have been consuming large doses of cannabis for several years. One of the major diagnostic criteria is the alleviation of symptoms by hot showers. The syndrome was first described in 2004 and is so far neither completely understood nor well known. The latter leads to continued morbidity in concerned subjects and unnecessary expenses for futile investigations. Standard treatments of vomiting as 5-HT3 or D2-receptor antagonists have been shown to be ineffective in alleviating the symptoms. The only long-term satisfying treatment option is the complete abstinence from cannabis consumption.* Case Summary.* In this case report we describe a 26-year-old male Caucasian long-term cannabis consumer who repeatedly presented in our emergency room with cyclic severe nausea and vomiting ceased by hot showers and resistant to all other treatments. The final diagnosis was not established until his third visit to the ER.* Conclusion.* CHS is an important differential diagnosis in patients who present with cyclic vomiting and abdominal pain with a history of long-term cannabis use. Recognition of this syndrome is important in order to avoid unnecessary clinical testing and to help the patients break the cycle of drug use.

## 1. Introduction

Cannabis is a commonly used recreational drug, which contains the principal psychoactive component delta 9-tetrahydrocannabinol (THC). Cannabis use is associated with several short- and long-term side effects such as changes in mood, impaired memory, impaired attention, depression, and anxiety and it is correlated with schizophrenia [1, 2]. However, cannabis use also has a therapeutic dimension due to its antiemetic and appetite-enhancing effects that is why it is sometimes used as a treatment for oncological patients suffering from nausea, vomiting, and loss of appetite during or after chemotherapy [3, 4]. The Cannabinoid Hyperemesis Syndrome is rare and was first described in the year 2004 by Allen et al. [5] and 2009 by Sontineni et al. [6] who first tried to establish diagnostic criteria. It has a delay in onset and is hence mostly observed in subjects who have been consuming large doses of cannabis over a period of several years. The syndrome can lead to complications like metabolic alkalosis, hypokalemia, acute kidney injury [7], or injuries of the oesophagus. Diagnostic criteria were renewed after a case series of 98 patients of Mayo Clinic in 2012 and include severe cyclic nausea and vomiting, resolution with cannabis cessation, relief of symptoms with hot showers or baths, abdominal pain, and weekly use of marijuana as major criteria [8] (see “Proposed Clinical Criteria by Simonetto et al. Mayo Clinic, 2012 [8]”). Faced with patients presenting the underdiagnosed CHS, physicians often perform ample and expensive investigations and workups that are futile in many cases. The only satisfying treatment is the complete abstinence from cannabis use.

*Proposed Clinical Criteria by Simonetto et al. Mayo Clinic, 2012 [8]*

Long-term cannabis use: essential for diagnosis
Major features:
  - Severe cyclic nausea and vomiting
  - Resolution with cannabis cessation
  - Relief of symptoms with hot showers or baths
  - Abdominal pain: epigastric our periumbilical
  - Weekly use of marijuana
Supportive features:
  - Age less than 50 years
  - Weight loss of >5 kg
  - Morning predominance of symptoms
  - Normal bowel habits
  - Negative laboratory and radiographic and endoscopic test results

## 2. Case Presentation

### 2.1. Personal History of the Patient

A 26-year-old male Caucasian patient presents three times to our emergency room with recurrent vomiting and colicky abdominal pain during the years 2014 and 2015. Every time he reports at least 20 episodes of vomiting a day and a strong abdominal pain (8/10) predominantly in the epigastric region. During the crises, he is unable to intake liquids or food out of fear of triggering vomiting. Episodes last about 24 to 48 hours and are refractory to all standard treatments for nausea and vomiting. His personal medical history reveals Gilbert's syndrome and a myringoplasty at the age of 5. The patient consumes cannabis twice a day since at least 10 years alongside an alcohol consumption which is reported to be irregular with 3 portions a week in average. He has been presenting these episodes in an irregular pattern for about 5 years with a duration of 1-2 days. The patient does not present fever and reports to have rather liquid stools, without meeting criteria for diarrhea. Before his hospitalization, several blood tests, enhanced abdominal CT-scan, and abdomen ultrasound have been performed ambulatory to investigate his symptoms. Blood tests, including complete blood count, glucose, basic metabolic panel, and pancreatic and hepatic enzymes, were normal. Radiological workup describes sludge and small gall stones within the gall bladder without evidence of acute cholecystitis. Considering the history with the atypical pain pattern and vomiting, the diagnosis of symptomatic gall stone disease was retained and a laparoscopic cholecystectomy was performed in August 2015. After an initial clinical improvement, the patient relapses and presents the same symptoms few weeks later, without any change in intensity.

### 2.2. Physical Examination and Other Tests

At our ER department, the patient had normal vital signs and no fever and physical examination was normal except for dehydration and light epigastric tenderness during abdominal examination. A neurological assessment was negative for any focal neurological deficit. Blood tests showed a slight leukocytosis (12 Giga/l) and slight hypokalemia (3.4 mmol/l). Hepatic profile showed typical signs of Gilbert's syndrome with an elevated total and unconjugated bilirubin without any other abnormalities. Stool cultures appeared to be negative. An esophagogastroduodenoscopy did not show any signs of ulceration or gastritis and the biopsies proved to be negative for a* Helicobacter pylori* infection. A cerebral MRI ruled out central causes of vomiting. A second abdominal CT-scan excluded any intra-abdominal pathology, especially no sign of superior mesenteric artery syndrome. Metabolic tests excluded diabetes mellitus, porphyria, and adrenal and thyroid diseases. The patient did not respond to standard treatments of nausea and vomiting as metoclopramide, ondansetron, or droperidol. The only effective measure to calm his abundant vomiting was hot showers or baths. The symptoms ceased right away after standing under hot water during his inpatient stay.

### 2.3. Treatment and Follow-Up

The patient was discharged from the hospital after two days with the recommendation to completely stop the consumption of cannabis. Follow-up nine months after discharge revealed that the patient had smoked cannabis again about two weeks after discharge in October 2015 with a relapse of symptoms and then never consumed cannabis again. He has been completely free of episodes of vomiting and abdominal pain since this time.

## 3. Discussion

Cannabis is of extraordinary cultural importance as it is the most commonly used illicit drug worldwide and ranks on the third place of recreational drugs behind alcohol and tobacco [9]. Every year about 130–230 million people use cannabis as a recreational drug [10] whereupon the percentage of legal and medical use is rising since a strong legalization movement has been established in people and governments of several countries over the last few years. It is therefore crucial to have solid evidence based information about all its possible health risks. There are many well explored short- and long-term side effects such as changes in mood, impaired memory, impaired attention, depression anxiety, and its correlation with schizophrenia [1, 2, 11].

The exact mechanisms of how and by which receptors the effects of THC are mediated in the human body are not yet completely understood. There are two main G-protein coupled receptors: the CB1 and CB2 receptor. The CB1 receptor is found in the central and peripheral nervous system, for example, in the hippocampus, the basal ganglia, and the cerebellum as well as in the enteric nervous system, and plays a neuromodulator role [12]. We know less about the CB2 receptor which is found primarily in immune cells and seems to have immunomodulatory effects [4, 13]. THC modulates the hypothalamus-pituitary axis by a diminution of secretion of anterior pituitary hormones like prolactin, growth hormone, thyroid hormone, or gonadotropin as well as an augmentation of corticotropin secretions [13]. These changes in the hypothalamus-pituitary-adrenal axis might also implicate changes in the thermoregulation. In a mouse model a decrease in body temperature has been described after the administration of THC [14] which might be an approach to a possible explanation of the compulsive bathing behavior in CHS patients.

The antiemetic effect of cannabis is mediated by an activation of CB-1 receptors in the brainstem and the enteric nervous system [15]. We still lack understanding of how a paradoxical hyperemetic effect can develop in long-term users, although several hypotheses were established during the last years. THC is lipophilic and accumulates in body fat, which serves as a long-term storage and probably liberates THC due to lipolysis in times of food deprivation and stress [13]. This might be one of the key aspects in the development of the CHS considering that mostly long-term users are concerned. However, since only a very small percentage of long-term cannabis consumers develop a CHS we need to identify risk factors for the development. Galli et al. proposed that the genetic polymorphisms in the cytochrome P450 enzymes and resulting in these possible excessive levels of emetogenic cannabinoid metabolites might be the cause [13].

Another important aspect is the quality of THC as a partial agonist on cannabinoid CB1 receptors. In case of chronic use and high tissue concentrations the antagonist nature of THC may emerge and induce a sudden withdrawal [16]. A sudden cessation from prolonged cannabis use might induce hyperemesis as already stated by Allen et al. in 2004 [5]. A downregulation of receptor density and desensitization of transduction mechanisms (tolerance) which can rapidly develop in case of chronic use [17] can also induce the transformation of THC from a partial agonist to an antagonist on CB1 receptors.

Eventually THC delays gastric emptying through the CB1 receptors found on the gastrointestinal system [18]. This effect has proven to be resistant to the development of tolerance [19].

Given the high prevalence of chronic cannabis abuse we presume that the syndrome is often underdiagnosed or misdiagnosed as cyclic vomiting or psychogenic vomiting. Further studies are warranted to better understand the pathophysiology and to identify risk factors as well as to validate the diagnostic criteria. Meanwhile, clinicians must evoke this diagnosis to avoid extensive workup and sometimes even futile surgery. Many of these patients relapse upon resuming cannabis because of their perception that it will have beneficial effects on nausea. Patient education should therefore be provided with emphasis on the paradoxical nature of the symptoms of CHS.

## 4. Conclusion

The Cannabinoid Hyperemesis Syndrome is a challenging differential diagnosis in patients who present with cyclic vomiting and abdominal pain with history of cannabis use. Recognition of this syndrome is important in order to avoid costly and unnecessary clinical testing and to help the patient understand and break the cycle of drug use. We emphasize the importance of a complete anamnestic inquiry concerning drug consumption and relief by hot showers or baths in those patients.

## Abbreviations

CHS: Cannabinoid Hyperemesis Syndrome
ER: Emergency room
5-HT3 receptor: 5-hydroxytryptamine 3 (serotonin) receptor
US: Ultrasound
THC: Delta 9-tetrahydrocannabinol.

## Additional Points

*Availability of Data and Materials.* The authors agree to make the raw data and materials described in our manuscript freely available.
